# Direction of Arrival Estimation in Elliptical Models via Sparse Penalized Likelihood Approach

**DOI:** 10.3390/s19102356

**Published:** 2019-05-22

**Authors:** Chen Chen, Jie Zhou, Mengjiao Tang

**Affiliations:** 1 College of Mathematics, Sichuan University, Chengdu 610064, China; chenchen_uni@foxmail.com; 2 Center for Information Engineering Science Research, School of Electronics and Information Engineering, Xi’an Jiaotong University, Xi’an 710049, China; qingqing_tmj@126.com

**Keywords:** direction of arrival (DOA), complex elliptically symmetric (CES) distributions, sparse penalized likelihood method, majorization-minimization (MM) algorithm

## Abstract

In this paper, an $l_{1}$-penalized maximum likelihood (ML) approach is developed for estimating the directions of arrival (DOAs) of source signals from the complex elliptically symmetric (CES) array outputs. This approach employs the $l_{1}$-norm penalty to exploit the sparsity of the gridded directions, and the CES distribution setting has a merit of robustness to the uncertainty of the distribution of array output. To solve the constructed non-convex penalized ML optimization for spatially either uniform or non-uniform sensor noise, two majorization-minimization (MM) algorithms based on different majorizing functions are developed. The computational complexities of the above two algorithms are analyzed. A modified Bayesian information criterion (BIC) is provided for selecting an appropriate penalty parameter. The effectiveness and superiority of the proposed methods in producing high DOA estimation accuracy are shown in numerical experiments.

## 1. Introduction

Estimating the directions of arrival (DOAs) of a number of far-field narrow-band source signals is an important problem in signal processing. Many DOA estimation methods were proposed early on, such as multiple signal classification (MUSIC) [1], estimation of signal parameters via rotation invariance techniques (ESPRIT) [2] and their variants [3,4]. Many of them work well when having accurate estimates of the array output covariance matrix and source number. In scenarios with sufficient array snapshots and a moderately high signal-to-noise (SNR), the array output covariance matrix and source number can be accurately estimated.

Recently, some sparse DOA estimation methods are popularly proposed based on sparse constructions of the array output model. They are applicable if the number of sources is unknown and many of them are effective when with a limited number of data snapshots. In [5], the sparse DOA estimation methods are categorized into three groups: the on-grid, the off-grid and the grid-less. The on-grid method, which is widely studied and straightforward to implement, assumes that the true DOAs are on a predefined grid [6,7,8,9,10,11,12,13,14,15,16,17,18,19,20,21,22]. The off-grid method also uses a prior grid but does not constrain the DOA estimates to be on this grid, while it introduces more unknown parameters to be estimated and complicates the algorithm [23,24,25,26,27,28]. The grid-less method directly operates in the entire direction domain without a pre-specified grid but, currently, is developed mainly for linear array and encounters rather large computational burden [29,30,31]. Although the on-grid methods may induce the grid mismatch, it is still attractive due to its easy accessibility in a wide range of applications (see, e.g., [28,32,33]). To alleviate the grid mismatch, methods for selecting an appropriate prior grid are proposed in [9,34].

During the past two decades, various on-grid sparse techniques, such as linear least-square, covariance-based and maximum-likelihood (ML) type methods, were researched. The linear least-square methods [6,9,11,12], minimizing the $l_{2}$-norm of the noise residual of the deterministic model, enforce the sparsity by constraining the $l_{p}$-norm of the signal vector for p∈[0,1] (note that $l_{p}$-norm for p∈[0,1) is defined as the same form as the one for p≥1 but is not truly a norm in mathematics). The covariance-based methods, such as the sparse iterative covariance-based estimation (SPICE) method [13,14] and its variants [15,16], and those proposed in [17,18,19,20,21,22], are derived from different covariance-fitting criteria and use the $l_{p}$-norm penalty to enforce the sparsity. The ML type methods, including the sparse Bayesian learning (SBL) methods [7,8,10] and the likelihood-based estimation of sparse parameters (LIKES) method [15,16], are deduced under the assumption that the array output signal is multivariate Gaussian distributed. The SBL methods use different prior distributions to model the sparsity. The LIKES method appears to be not explicitly utilizing the sparsity, but provides sparse estimates.

The ML principle is generally believed to be statistically more sound than the covariance-fitting principle [15]. The existing on-grid ML methods are developed on the Gaussian distribution assumption that are not satisfied in many signal processing practical applications. The complex elliptically symmetric (CES) distributions, containing the *t*-distribution, the *K*-distribution, the Gaussian distribution and so on, can be used to characterise the Gaussian and non-Gaussian random variables. Particularly, the heavy-tailed data, which usually appear in the field of radar and array signal processing [35,36], can be modeled by some CES distributions.

The $l_{p}$-norm penalty with p∈[0,1] is well-known for its contributions in deriving sparse solutions [37]. In particular, the convex $l_{1}$-norm penalty, also known as the least absolute shrinkage and selection operator (LASSO) penalty, is easy to handle and widely used in various applications. Generally, a sparse and accurate estimate of the unknown sparse parameter can be expected when minimizing the negative likelihood plus an $l_{p}$-norm penalty.

For estimating the DOAs from the CES distributed array outputs, we provide an $l_{1}$-norm penalized ML method based on a sparse reconstruction of the array output covariance matrix. The characteristics and advantages of our method are explained as follows:

1. Our method is a sparse on-grid method based on the penalized ML principle, and is designed especially for the CES random output signals. The Gaussian distribution and many heavy-tailed and light-tailed distributions are included in the class of the CES distributions, and it is worth mentioning that the heavy-tailed output signals that are non-Gaussian are common in the field of signal processing [35,36].
2. The sparsity of the unknown sparse vector is enforced by penalizing its $l_{1}$-norm. When the penalty parameter becomes zero, the proposed method is actually the general ML DOA estimation method that is applicable to the scenarios with CES random output signals.
3. Two penalized ML optimization problems are formulated for spatially uniform and non-uniform white sensor noise, respectively. Since it is difficult to solve the two non-convex penalized ML optimizations globally, two majorization-minimization (MM) algorithms having different iterative procedures are developed for seeking the optimal solutions locally for each of them. Some discussions on the computational complexities of the above two algorithms are provided. In addition, the optimal penalty parameter is suggested.
4. The proposed methods are evaluated numerically in scenarios with Gaussian and non-Gaussian output signals. Particularly, the performance gains originated from the added $l_{1}$-norm penalty are numerically demonstrated.

The remainder of this paper is organized as follows. Section 2 introduces a sparse CES data model of the array output. In Section 3, a sparse penalized ML method is developed to estimate the DOAs. For solving the proposed $l_{1}$-penalized ML optimizations that are non-convex, algorithms in the MM framework are developed in Section 4. Section 5 numerically shows the performance of our method in Gaussian and non-Gaussian scenarios. Finally, some conclusions are given in Section 6.

### Notations

The notation $C^{m}\left(R^{m}\right)$ denotes the set of all *m*-dimensional complex- (real-) valued vectors, and $C^{m\times n}\left(R^{m\times n}\right)$ denotes the set of all m×n complex- (real-) valued matrices. $1_{p}$ and $0_{p}$ are the *p*-dimensional vectors with all elements equal to 1 and 0, respectively. $I_{p}$ is the p×p identity matrix. ${∥\cdot ∥}_{1}$ and ${∥\cdot ∥}_{2}$ denote the $l_{1}$-norm and $l_{2}$-norm of a vector, respectively. The superscripts ${\left(\cdot \right)}^{T}$ and ${\left(\cdot \right)}^{H}$, respectively, denote the transpose and the conjugate transpose of a vector or matrix. The imaginary unit is denoted as *ı* defined by ${ı}^{2}=-1$.

For a vector $x={\left[x_{1},\ldots ,x_{p}\right]}^{T}\in C^{p}$, define element-wise square root function $\mathrm{sqrt}\left(x\right)={\left[\sqrt{|x_{1}|},\ldots ,\sqrt{|x_{p}|}\right]}^{T}$ and element-wise division operation $x⊘y={\left[x_{1}/y_{1},\ldots ,x_{p}/y_{p}\right]}^{T}$ for a vector $y={\left[y_{1},\ldots ,y_{p}\right]}^{T}\in C^{p}$ with non-zero elements. $\max\left(x\right)=\max\left\{x_{1},\ldots ,x_{p}\right\}$, $x_{\left[a:b\right]}={\left[x_{a},\ldots ,x_{b}\right]}^{T}$ for 1≤a≤b≤p, x>0(≥0) means $x_{i}>0\;\left(\geq 0\right)$ for i=1,…,p, [x;z] denotes the stacked vector of the column vectors x and z. Diag(x) denotes a square diagonal matrix with the elements of vector x on the main diagonal, and ${\left(\mathrm{Diag}\left(x\right)\right)}^{-1}$ denotes the diagonal matrix with main diagonal elements $1/x_{i}\;\left(i=1,\ldots ,p\right)$ by taking 1/0=∞.

For a square matrix $X\in C^{p\times p}$, $X_{i,j}$ denotes the (i,j)th entry of X, X>0(≥0) means that X is Hermitian and positive definite (semidefinite), tr(X) denotes the trace of X, and diag(X) denotes a column vector of the main diagonal elements of X.

## 2. Problem Formulation

Consider the problem of estimating the DOAs of $k_{0}$ narrow-band signals impinging on an array of *m* sensors.

Given a set of grid points

(1) $$\{\theta_{1},\cdots ,\theta_{k}\},$$

we assume that the true $k_{0}$ ($k_{0}\ll k$) DOAs, respectively, denoted as $\xi_{1},\cdots ,\xi_{k_{0}}$, take values in it. The array output measurement at the instant *t*, denoted as $x\left(t\right)\in C^{m}$, can be modeled as

(2) x(t)=A(θ)s(t)+v(t),

where

- $\theta ={\left[\theta_{1},\ldots ,\theta_{k}\right]}^{T}$,
- $A\left(\theta \right)=\left[a\left(\theta_{1}\right),\ldots ,a\left(\theta_{k}\right)\right]\in C^{m\times k}$ is the known array manifold matrix with $a(\theta_{i})$ being the steering vector corresponding to $\theta_{i}$, i=1,…,k;
- $s\left(t\right)={\left[s_{1}\left(t\right),\ldots ,s_{k}\left(t\right)\right]}^{T}\in C^{k}$ is the source signal vector at the time instant *t*, in which $s_{i}\left(t\right)$ is the unknown random signal from a possible source at $\theta_{i}$ and then is zero if $\theta_{i}$ is not in the true DOA set $\left\{\xi_{1},\cdots ,\xi_{k_{0}}\right\}$, i=1,…,k;
- $v\left(t\right)={\left[v_{1}\left(t\right),\ldots ,v_{m}\left(t\right)\right]}^{T}\in C^{m}$ is the noise vector impinging on the sensor array at the time instant *t*.

Some necessary statistical assumptions are made as follows:

- The possible source signals $s_{1}\left(t\right),\ldots ,s_{k}\left(t\right)$ are uncorrelated and zero-mean at any time instant *t*.
- The noise components $v_{1}\left(t\right),\ldots ,v_{m}\left(t\right)$ are uncorrelated, zero-mean, and independent of $s_{1}\left(t\right),\ldots ,s_{k}\left(t\right)$ for any time instant *t*.
- The n(n>m) snapshots x(1),…,x(n) of sensor array signals are independent and identically distributed from a CES distribution.

Note that the zero-mean assumptions above are common in the signal processing literature [5,35]. The CES distribution setting of the array output x(t) enables us to effectively process the Gaussian, heavy-tailed or light-tailed array snapshots, because the class of CES distributions [38] includes the Gaussian distribution, the *t*-distribution, the *K*-distribution and so on.

For the simplicity of the notations, we denote A(θ) as A and $a(\theta_{i})$ as $a_{i}$ for i=1,…,k in the following. Under the above assumptions, we can find that the array output x(t) in Equation (2) at any time instant *t* has mean zero and covariance matrix

(3) $$\begin{array}{c} R=E\left[x\left(t\right)x{\left(t\right)}^{H}\right]=APA^{H}+V, \end{array}$$

where

(4) $$\begin{array}{c} P=E[s\left(t\right)s{\left(t\right)}^{H}]=\mathrm{Diag}\left(p\right) \end{array}$$

is the (unknown) source signal covariance matrix with signal power vector $p={\left[p_{1},\ldots ,p_{k}\right]}^{T}$, and

(5) $$\begin{array}{c} V=E[v\left(t\right)v{\left(t\right)}^{H}]=\mathrm{Diag}\left(\sigma \right) \end{array}$$

is the (unknown) noise covariance matrix with the noise power vector $\sigma ={\left[\sigma_{1}^{2},\ldots ,\sigma_{m}^{2}\right]}^{T}$. The matrix R can be rewritten as

(6) $$\begin{array}{c} R=\left[A,I_{m}\right]\mathrm{Diag}\left(\left[p;\sigma \right]\right){\left[A,I_{m}\right]}^{H}. \end{array}$$

For any i=1,…,k, the signal power $p_{i}=0$ if $\theta_{i}$ is not in the set of the true DOAs. Therefore, the true DOAs can be identified by the locations of nonzero elements of the power vector p. In the following, the DOA estimation problem is formulated as a problem of estimating the locations of nonzero elements of the power vector p.

## 3. Sparse DOA Estimation

The array output x(t) in Equation (2) is CES distributed with mean zero and covariance matrix R, and then the normalized random vector

(7) $$\begin{array}{c} y\left(t\right)≜x\left(t\right)/{∥x\left(t\right)∥}_{2}, \end{array}$$

which actually refers to the angle of the array output vector x(t), has a complex angular central Gaussian (ACG) distribution [35] with the probability density function

(8) $$\begin{array}{c} p\left(y\left(t\right);R\right)\propto \det{\left(R\right)}^{-1}{\left(y{\left(t\right)}^{H}R^{-1}y\left(t\right)\right)}^{-m}. \end{array}$$

Denote

(9) $$\begin{array}{c} L_{0}\left(R\right)=\log\det\left(R\right)+\frac{m}{n}\sum_{t=1}^{n}\log\left(x{\left(t\right)}^{H}R^{-1}x\left(t\right)\right), \end{array}$$

and then the negative log-likelihood of $y\left(t\right)=x\left(t\right)/{∥x\left(t\right)∥}_{2},\;t=1,\ldots ,n$, becomes

(10) $$\begin{array}{c} nL_{0}\left(R\right)-m\sum_{t=1}^{n}{\log(∥x\left(t\right)∥}_{2}^{2})+c_{1}, \end{array}$$

where $c_{1}$ is a constant.

### 3.1. Spatially Non-Uniform White Noise

In the case of spatially non-uniform white sensor noise, not all noise variances $\sigma_{i}^{2}$, i=1,⋯,m, are equal. Assuming $\sigma_{m}^{2}>0$, we denote

(11) $$\begin{array}{cc} r & =\left[p;\sigma_{\left[1:m-1\right]}\right]/\sigma_{m}^{2}, \end{array}$$

(12) $$\begin{array}{cc} W & =B\mathrm{Diag}\left(r\right)B^{H}+J_{m}, \end{array}$$

where $B\in C^{m\times (k+m-1)}$ is the first (k+m−1) columns of $\left[A,I_{m}\right]$ and $J_{m}$ is an m×m matrix with the (m,m)-entry being 1 and the other entries being 0. Since

(13) $$\begin{array}{cc} W & =R/\sigma_{m}^{2}, \end{array}$$

(14) $$\begin{array}{cc} L_{0}\left(aR\right) & =L_{0}\left(R\right),\forall a>0 \end{array}$$

and that the locations of nonzero elements of the sparse vector $r_{\left[1:k\right]}$ identify the true DOAs, we formulate the DOA estimation problem as solving the penalized likelihood optimization problem

(15) $$\underset{r\in \Omega }{\arg\min}L_{0}\left(W\right)+\lambda {∥r_{\left[1:k\right]}∥}_{1},$$

where

(16) $$\Omega =\{t\in R^{k+m-1}|t\geq 0,B\mathrm{Diag}\left(t\right)B^{H}+J_{m}>0\}$$

and λ≥0 is pre-specified. The $l_{1}$-norm penalty term $\lambda ∥r_{\left[1:k\right]}{∥}_{1}$ would help deduce a sparse solution, and the penalty parameter λ controls the sparsity level [37].

**Remark** **1.** *We explain why the DOAs are not estimated by solving the plausible $l_{1}$-penalized ML optimization problem*

(17) $$\begin{array}{c} \underset{p,\sigma }{\arg\min}L_{0}\left(R\right)+\lambda {∥p∥}_{1}. \end{array}$$

*Recalling Equation (6), we find for any $\lambda_{1}>0$ and $\lambda_{2}>0$,*

(18) $$\begin{array}{c} \begin{array}{c} L_{0}\left(R\right)+\lambda_{1}{∥p∥}_{1}=L_{0}\left(\left[A,I_{m}\right]\mathrm{Diag}\left(\left[\frac{\lambda_{1}}{\lambda_{2}}p;\frac{\lambda_{1}}{\lambda_{2}}\sigma \right]\right){\left[A,I_{m}\right]}^{H}\right)+\lambda_{2}{∥\frac{\lambda_{1}}{\lambda_{2}}p∥}_{1}. \end{array} \end{array}$$

*Thus, [p,σ] is a locally optimal solution of Equation (17) with $\lambda =\lambda_{1}$ if and only if $\left[\frac{\lambda_{1}}{\lambda_{2}}p;\frac{\lambda_{1}}{\lambda_{2}}\sigma \right]$ is a locally optimal solution of Equation (17) with $\lambda =\lambda_{2}$. This means if we estimate p by Equation (17), the parameter λ cannot work theoretically in adjusting the sparsity level of the estimate of p. Instead of considering Equation (17), we formulate the optimization (Equation (15)) for the DOA estimation. In Equation (15), noticing the constant matrix $J_{m}$ in Equation (12), we find that different values of λ would result in solutions with different sparsity levels.*

### 3.2. Spatially Uniform White Noise

In the case of spatially uniform white sensor noise, $\sigma_{1}=\cdots =\sigma_{m}=\sigma$. Assuming $\sigma^{2}>0$, we denote

(19) $$\begin{array}{cc} q & =p/\sigma^{2}, \end{array}$$

(20) $$\begin{array}{cc} Q & =A\mathrm{Diag}\left(q\right)A^{H}+I_{m}. \end{array}$$

Estimating the DOAs means identifying the locations of nonzero elements of the vector q. Considering $Q=R/\sigma^{2}$, we can estimate q through solving the penalized likelihood optimization problem

(21) $$\underset{q\geq 0}{\arg\min}L_{0}\left(Q\right)+\lambda {∥q∥}_{1}$$

with λ≥0, where the term ${\lambda ∥q∥}_{1}$ plays the same role as the penalty term in Equation (15).

Note that the number of unknown parameters to be estimated is *k* in the case of spatially uniform noise in contrast to k+m−1 in the case of spatially non-uniform noise.

## 4. DOA Estimation Algorithms

In this section, we provide methods to solve the optimization problems in Equations (15) and (21) with λ fixed. As Equations (15) and (21) are non-convex, it is generally hard to give their globally optimal solutions. Based on the MM framework [39,40], we develop algorithms to find the locally optimal solutions of Equations (15) and (21).

A function f(x) is said to be majorized by a function $g(x|x_{0})$ at $x_{0}$, if $f\left(x\right)\leq g\left(x|x_{0}\right)$ for all *x* and $f\left(x_{0}\right)=g\left(x_{0}|x_{0}\right)$. In the MM framework, the problem $\arg\min_{x}f\left(x\right)$ can be solved through iteratively solving $x_{u+1}=\arg\min_{x}g\left(x|x_{u}\right)$.

When solving the problems in Equations (15) and (21) in the MM framework, majorizing functions can be constructed based on the following two inequalities. For any positive definite matrices $U\in C^{m\times m}$ and $U_{u}\in C^{m\times m}$ [40],

(22) $$\log\det\left(U\right)\leq \log\det\left(U_{u}\right)+\mathrm{tr}\left(U_{u}^{-1}U\right)-m$$

and

(23) $$\log\left(x{\left(t\right)}^{H}U^{-1}x\left(t\right)\right)\leq \log\left(x{\left(t\right)}^{H}U_{u}^{-1}x\left(t\right)\right)+\frac{x{\left(t\right)}^{H}U^{-1}x\left(t\right)}{x{\left(t\right)}^{H}U_{u}^{-1}x\left(t\right)}-1,$$

where both equalities are achieved at $U=U_{u}$.

### 4.1. Algorithms for Spatially Non-Uniform White Noise

In this subsection, using two different majorizing functions of the objection function in Equation (15), we develop two different MM algorithms named MM1 and MM2 to solve Equation (15).

#### 4.1.1. MM1 Algorithm

Denote

(24) $$W_{u}=B\mathrm{Diag}\left(r_{u}\right)B^{H}+J_{m},$$

where $r_{u}\in \Omega$. Replacing the U and $U_{u}$ in Equations (22) and (23) by W and $W_{u}$, respectively, we have for any $W>0,W_{u}>0$,

(25) $$\begin{array}{cc} L_{0}\left(W\right) & \leq \mathrm{tr}\left(W_{u}^{-1}W\right)+\frac{m}{n}\sum_{t=1}^{n}\frac{x{\left(t\right)}^{H}W^{-1}x\left(t\right)}{x{\left(t\right)}^{H}W_{u}^{-1}x\left(t\right)}+c_{2} \end{array}$$

(26) $$\begin{array}{c} =\mathrm{tr}\left(W_{u}^{-1}W\right)+\mathrm{tr}\left(M_{u}W^{-1}\right)+c_{2} \end{array}$$

where $c_{2}$ is a constant, the equality in Equation (25) is achieved at $W=W_{u}$, and

(27) $$M_{u}=\frac{m}{n}\sum_{t=1}^{n}\frac{x\left(t\right)x{\left(t\right)}^{H}}{x{\left(t\right)}^{H}W_{u}^{-1}x\left(t\right)}.$$

Denote the sum of the first two terms on the right side of Equation (26) and the $l_{1}$-norm penalty term in Equation (15) as $g_{1}\left(r|r_{u},\lambda \right)$, and then due to Equation (12), $g_{1}\left(r|r_{u},\lambda \right)$ can be rewritten as

(28) $$g_{1}\left(r|r_{u},\lambda \right)=\mathrm{tr}\left(M_{u}W^{-1}\right)+w_{u}^{T}r$$

with

(29) $$\begin{array}{cc} w_{u} & =\mathrm{diag}\left(B^{H}W_{u}^{-1}B\right)+\left[\lambda 1_{k};0_{m-1}\right]. \end{array}$$

From Equation (25), $g_{1}\left(r|r_{u},\lambda \right)+c_{2}$ is found to be a majorizing convex function of the objective function in Equation (15). Based on the MM framework, the problem in Equation (15) can be solved by iteratively solving the convex optimization problem

(30) $$r_{u+1}=\underset{r\in \Omega }{\arg\min}g_{1}\left(r|r_{u},\lambda \right).$$

**Proposition** **1**  (The MM1 algorithm for Equation (15)). *The sequence $\left\{r_{u}\right\}$ generated by*

(31) $$r_{u+1}=\underset{r\geq 0}{\arg\min}g_{1}\left(r|r_{u},\lambda \right)$$

*with any initial value $r_{0}\in \Omega$ converges to a locally optimal solution of the optimization problem in Equation (15).*

**Proof.**  Since $g_{1}\left(r|r_{u},\lambda \right)$ tends to +∞ as W goes to the boundary of the positive semidefinite cone, the optimization problems in Equations (30) and (31) are equivalent.

This proposition follows by the convergence property of the MM algorithm [40] and the fact that $L_{0}\left(W\right)\to +\infty$ with probability 1 as W tends to the boundary of the positive semidefinite cone [41].  □

Due to Equation (11), $r_{0}={\left[{\left(r_{1}\right)}_{0},\ldots ,{\left(r_{k+m-1}\right)}_{0}\right]}^{T}$ required in the iteration in Equation (31) can be the one obtained by inserting the initial estimate of [p;σ] presented in [13]. Specifically,

(32) $$\begin{array}{c} {\left(r_{i}\right)}_{0}=\frac{b_{i}^{H}\hat{R}b_{i}}{{\hat{R}}_{m,m}{∥b_{i}∥}_{2}^{4}}>0,\;i=1,\ldots ,k+m-1, \end{array}$$

where $b_{i}$ is the *i*th column of the matrix B and

(33) $$\begin{array}{c} \hat{R}=\frac{1}{n}\sum_{t=1}^{n}x\left(t\right)x{\left(t\right)}^{H}. \end{array}$$

To solve the optimization problem in Equation (31) globally, we develop two available solvers: the coordinate descent (CD) and SPICE-like solvers. Denote $r={\left[r_{1},\cdots ,r_{k+m-1}\right]}^{T}$ in the following.

**Proposition** **2**  (The CD solver for Equation (31)). *The sequence of r generated by iterating*

(34) $$r_{i}=\left(\left(\sqrt{\frac{\beta_{i}}{\alpha_{i}}}-1\right)\frac{1}{\gamma_{i}}\right)\delta \left(\beta_{i}-\alpha_{i}\right),\;i=1,\cdots ,k+m-1$$

*with any initial value r≥0 converges to the globally optimal solution $r_{u+1}$ of the problem in Equation (31), where δ(·) is the indicator function of the interval (0,∞),*

(35) $$\begin{array}{cc} \alpha_{i} & ={\left(w_{i}\right)}_{u}, \end{array}$$

(36) $$\begin{array}{cc} \beta_{i} & =b_{i}^{H}N_{i}^{-1}M_{u}N_{i}^{-1}b_{i}, \end{array}$$

(37) $$\begin{array}{cc} \gamma_{i} & =b_{i}^{H}N_{i}^{-1}b_{i}, \end{array}$$

*with ${\left(w_{i}\right)}_{u}$ being the ith element of the vector $w_{u}$, and *

(38) $$N_{i}=\sum_{l\neq i}r_{l}b_{l}b_{l}^{H}+J_{m}.$$

**Proof.**  By the general analysis of the CD method in [42], it is easy to find that the convex problem in Equation (31) can be globally solved by iterating

(39) $$r_{i}=\underset{r_{i}\geq 0}{\arg\min}g_{1}\left(r|r_{u},\lambda \right),\;i=1,\ldots ,k+m-1$$

until convergence.

Due to

(40) $$W=B\mathrm{Diag}\left(r\right)B^{H}+J_{m}=r_{i}b_{i}b_{i}^{H}+N_{i}$$

and

(41) $${\left(r_{i}b_{i}b_{i}^{H}+N_{i}\right)}^{-1}=N_{i}^{-1}-\frac{r_{i}N_{i}^{-1}b_{i}b_{i}^{H}N_{i}^{-1}}{1+r_{i}b_{i}^{H}N_{i}^{-1}b_{i}},$$

the iteration in Equation (39) can be reformulated as

(42) $$r_{i}=\underset{r_{i}\geq 0}{\arg\min}\alpha_{i}r_{i}-\frac{\beta_{i}r_{i}}{1+\gamma_{i}r_{i}},\;i=1\ldots ,k+m-1.$$

In addition, $\alpha_{i}>0,\beta_{i}>0,\gamma_{i}>0$ for i=1,…,k+m−1. Solving the convex optimization problems in Equation (42) by the gradient method, we conclude that the equations in Equation (42) are equivalent to those in Equation (34). □

As the solver in Proposition 2 is derived by the CD method, we call it the CD solver. The MM1 algorithm in Proposition 1 with the CD solver is specially named the MM1-CD algorithm.

For clarity, detailed steps of the MM1-CD algorithm for Equation (15) are presented in Algorithm 1.

Note that, in the CD solver, for i=1,…,k, we have

(43) $$\beta_{i}=\frac{m}{n}\sum_{t=1}^{n}\zeta_{i}\zeta_{i}^{H}$$

where

(44) $$\zeta_{i}=\frac{a_{i}^{H}N_{i}^{-1}x\left(t\right)}{\sqrt{x{\left(t\right)}^{H}W_{u}^{-1}x\left(t\right)}}$$

with $a_{i}$ being the *i*th column of the matrix A. The $\beta_{i}$ can be interpreted as the correlation between the signal from a possible source at the grid $\theta_{i}$ and the array responses x(1),…,x(n). From the indicator function $\delta (\beta_{i}-\alpha_{i})$ in Equation (34), we find that it is more likely to force to zeros the powers of the assumed signals that are less correlated with the array responses.

**Proposition 3**  (The SPICE-like solver for Equation (31)). *The sequence of r generated by iterating*

(45) $$r=\mathrm{sqrt}(\mathrm{diag}\left(\mathrm{Diag}\left(r\right)B^{H}W^{-1}M_{u}W^{-1}B\mathrm{Diag}\left(r\right)\right)⊘w_{u})$$

*with any initial value r>0 converges to the globally optimal solution $r_{u+1}$ of problem in Equation (31).*

**Algorithm 1** The MM1-CD algorithm for spatially non-uniform white noise.
1:Give $r_{0}$ and ϵ,2:u=0,3:
**repeat**
4:    $W_{u}=B\mathrm{Diag}\left(r_{u}\right)B^{H}+J_{m}$,5:    Calculate $M_{u}$ and $w_{u}$,6:    d=0,7:    $r^{0}=r_{u}$, $r=r^{0}$,8:    **repeat**9:        **for** i=1:k+m−1 **do**10:           $N_{i}=\sum_{l\neq i}r_{l}b_{l}b_{l}^{H}+J_{m}$,11:           Calculate $\alpha_{i}$, $\beta_{i}$ and $\gamma_{i}$,12:           **if** $\beta_{i}\leq \alpha_{i}$ **then**13:               $r_{i}=0$,14:           **else**15:               $r_{i}=\left(\sqrt{\beta_{i}/\alpha_{i}}-1\right)/\gamma_{i}$,16:           **end if**17:        **end for**18:        d=d+1,19:        $r^{d}={\left[r_{1},\ldots ,r_{k+m-1}\right]}^{T}$,20:    **until** $∥r^{d}-r^{d-1}{∥}_{2}/{∥r^{d-1}∥}_{2}<\epsilon$,21:    u=u+1,22:    $r_{u}=r^{d}$,23:**until**$∥r_{u}-r_{u-1}{∥}_{2}/{∥r_{u-1}∥}_{2}<\epsilon$.

**Proof.**  It is obvious that Equation (31) and the SPICE criterion in [15] are with similar forms. By the same way as the SPICE criterion is analyzed in [15], we find that the globally optimal solution of the problem in Equation (31) is identical to the minimizer r of the optimization problem

(46) $$\begin{array}{c} \begin{array}{cc} \min_{r\geq 0,E\in C^{(k+m)\times n}}\; & \mathrm{tr}\left(E^{H}{\left(\mathrm{Diag}\left(\left[r;1\right]\right)\right)}^{-1}E\right)+w_{u}^{T}r\\ s.t.\; & \left[A,I_{m}\right]E=X_{u}, \end{array} \end{array}$$

where $X_{u}=\left[\tilde{x}\left(1\right),\ldots ,\tilde{x}\left(n\right)\right]$ with

(47) $$\tilde{x}\left(t\right)=\sqrt{\frac{m}{n}}\frac{x(t)}{\sqrt{x{\left(t\right)}^{T}W_{u}^{-1}x\left(t\right)}},\;t=1,\ldots ,n.$$

For a fixed r, the matrix E minimizing Equation (46) can be verified to be [15]

(48) $$\begin{array}{c} E=\mathrm{Diag}\left(\left[r;1\right]\right){\left[A,I_{m}\right]}^{H}W^{-1}X_{u}, \end{array}$$

and for a fixed E, the vector r minimizing Equation (46) can be readily given by

(49) $$\begin{array}{c} r=\mathrm{sqrt}({\left(\mathrm{diag}\left(EE^{H}\right)\right)}_{\left[1:k+m-1\right]}⊘w_{u}). \end{array}$$

The sequences of E and r, generated by alternately iterating Equations (48) and (49) from r>0, converge to the globally optimal solution of the convex problem in Equation (46) [14,15].

Due to $X_{u}X_{u}^{H}=M_{u}$, iterating Equation (49) is just iterating Equation (45). Thus, the sequence of r generated by Equation (45) converges to the minimizer r of Equation (46). □

The solver in Proposition 3 is named the SPICE-like solver, and the MM1 algorithm in Proposition 1 with it is called the MM1-SPICE algorithm. Algorithm 2 summarily illustrates the MM1-SPICE algorithm for Equation (15).

**Algorithm 2** The MM1-SPICE algorithm for spatially non-uniform white noise.
1:Give $r_{0}$ and ϵ,2:u=0,3:
**repeat**
4:    $W_{u}=B\mathrm{Diag}\left(r_{u}\right)B^{H}+J_{m}$,5:    Calculate $M_{u}$ and $w_{u}$,6:    d=0,7:    $r^{0}=r_{u}$,8:    **repeat**9:        $W^{d}=B\mathrm{Diag}\left(r^{d}\right)B^{H}+J_{m}$,10:        $r^{d+1}=\mathrm{sqrt}\left(\mathrm{diag}\left(\mathrm{Diag}\left(r^{d}\right)B^{H}{\left(W^{d}\right)}^{-1}M_{u}{\left(W^{d}\right)}^{-1}B\mathrm{Diag}\left(r^{d}\right)\right)⊘w_{u}\right)$,11:        d=d+1,12:    **until** $∥r^{d}-r^{d-1}{∥}_{2}/{∥r^{d-1}∥}_{2}<\epsilon$,13:    u=u+1,14:    $r_{u}=r^{d}$,15:**until**$∥r_{u}-r_{u-1}{∥}_{2}/{∥r_{u-1}∥}_{2}<\epsilon$.


From Propositions 1–3, it is found that the proposed MM1 algorithm is by an inner–outer iteration loop. The MM1-CD and the MM1-SPICE algorithms are with the identical outer loop but different nested inner loops. In addition, relationship and difference between the MM1-CD and the MM1-SPICE are discussed in Section 4.3.

#### 4.1.2. MM2 Algorithm

When $r_{u}>0$, it is found from [36] that, for any r>0,

(50) $$\left[\begin{array}{cc} C_{u}{\left(\mathrm{Diag}\left(\left[r;1\right]\right)\right)}^{-1}C_{u}^{H} & I_{m}\\ I_{m} & W \end{array}\right]=F^{H}F\geq 0,$$

where

(51) $$\begin{array}{cc} C_{u} & =W_{u}^{-1}\left[A,I_{m}\right]\mathrm{Diag}\left(\left[r_{u};1\right]\right), \end{array}$$

(52) $$\begin{array}{cc} F & =[{\left(\mathrm{Diag}\left(\left[r;1\right]\right)\right)}^{-\frac{1}{2}}C_{u}^{H},{\left(\mathrm{Diag}\left(\left[r;1\right]\right)\right)}^{\frac{1}{2}}{\left[A,I_{m}\right]}^{H}]. \end{array}$$

From Equation (50), we have

(53) $$\begin{array}{c} W^{-1}\leq C_{u}{\left(\mathrm{Diag}\left(\left[r;1\right]\right)\right)}^{-1}C_{u}^{H} \end{array}$$

with the equality achieved at $r=r_{u}$. The inequality in Equation (53) is also valid for any r∈Ω due to W>0.

Denote

(54) $$g_{2}\left(r|r_{u},\lambda \right)=\mathrm{tr}\left(C_{u}^{H}M_{u}C_{u}{\left(\mathrm{Diag}\left(\left[r;1\right]\right)\right)}^{-1}\right)+w_{u}^{T}r.$$

It is clear from Equation (53) that, when $r_{u}>0$, for any r∈Ω,

(55) $$g_{1}\left(r|r_{u},\lambda \right)\leq g_{2}\left(r|r_{u},\lambda \right),$$

where the equality is achieved at $r=r_{u}$. Therefore, at any $r_{u}>0$, $g_{2}\left(r|r_{u},\lambda \right)+c_{2}$ majorizes the objective function in Equation (15) for r∈Ω.

**Proposition** **4**  (The MM2 algorithm for Equation (15)). *The sequence $\left\{r_{u}\right\}$ generated by*

(56) $$r_{u+1}=\mathrm{sqrt}\left(\mathrm{diag}\left(\mathrm{Diag}\left(r_{u}\right)B^{H}W_{u}^{-1}M_{u}W_{u}^{-1}B\mathrm{Diag}\left(r_{u}\right)\right)⊘w_{u}\right)$$

*with any initial value $r_{0}>0$ converges to a locally optimal solution of the problem in Equation (15).*

**Proof.**  Through the convergence analysis in [36], we have that, although  Equation (55) is valid only when $r_{u}>0$, the sequence $\left\{r_{u}\right\}$ generated

(57) $$\begin{array}{c} r_{u+1}=\underset{r\in \Omega }{\arg\min}g_{2}\left(r|r_{u},\lambda \right) \end{array}$$

with any initial value $r_{0}>0$ converges to a locally optimal solution of the problem in Equation (15). It is worth mentioning that the elements of the coefficient vector $\mathrm{diag}(C_{u}^{H}M_{u}C_{u})$ in Equation (54) are positive. By solving the optimization problem in Equation (57) using the gradient method, the iteration procedure in Equation (57) is found to be exactly Equation (56).  □

The $r_{0}$ involved in the iteration procedure in Equation (56) can be the one given in Equation (32). Summarily, Algorithm 3 gives the detailed steps of the MM2 algorithm for the problem in Equation (15).

**Algorithm 3** The MM2 algorithm for spatially non-uniform white noise.
1:Give $r_{0}$ and ϵ,2:u=0,3:
**repeat**
4:    $W_{u}=B\mathrm{Diag}\left(r_{u}\right)B^{H}+J_{m}$,5:    Calculate $M_{u}$ and $w_{u}$,6:    $r_{u+1}=\mathrm{sqrt}\left(\mathrm{diag}\left(\mathrm{Diag}\left(r_{u}\right)B^{H}W_{u}^{-1}M_{u}W_{u}^{-1}B\mathrm{Diag}\left(r_{u}\right)\right)⊘w_{u}\right)$,7:    u=u+1,8:**until**$∥r_{u}-r_{u-1}{∥}_{2}/{∥r_{u-1}∥}_{2}<\epsilon$.


### 4.2. DOA Estimation for Spatially Uniform White Noise

The DOA estimation in the case of spatially uniform white noise amounts to solving the optimization problem in Equation (21). Problems in Equations (21) and (15) are with similar forms, but Equation (21) involves a smaller number of unknown parameters. By the same way as the problem in Equation (15) is analyzed in Section 4.1, we can naturally derive the algorithms to solve Equation (21). The algorithms for Equation (21) are still named MM1 (including MM1-CD and MM1-SPICE) and MM2.

#### 4.2.1. MM1 Algorithm

**Proposition** **5**  (The MM1 algorithm for Equation (21)). *Denote*

(58) $$\begin{array}{c} h_{1}\left(q|q_{u},\lambda \right)=\mathrm{tr}\left(Z_{u}Q^{-1}\right)+e_{u}^{T}q \end{array}$$

*with*

(59) $$\begin{array}{cc} Z_{u} & =\frac{m}{n}\sum_{t=1}^{n}\frac{x\left(t\right)x{\left(t\right)}^{H}}{x{\left(t\right)}^{H}Q_{u}^{-1}x\left(t\right)}, \end{array}$$

(60) $$\begin{array}{cc} e_{u} & =\mathrm{diag}\left(A^{H}Q_{u}^{-1}A\right)+\lambda 1_{k}, \end{array}$$

(61) $$\begin{array}{cc} Q_{u} & =A\mathrm{Diag}\left(q_{u}\right)A^{H}+I_{m}, \end{array}$$

*and then the sequence $\left\{q_{u}\right\}$ generated by*

(62) $$\begin{array}{c} q_{u+1}=\underset{q\geq 0}{\arg\min}h_{1}\left(q|q_{u},\lambda \right) \end{array}$$

*with any initial value q≥0 converges to a locally optimal solution of the problem in Equation (21).*

To solve the optimization problem in Equation (62), using the same method as Equation (31) is solved, we offer two different iterative solvers for Equation (62).

**Proposition** **6**  (The CD solver for Equation (62)). *The sequence of q generated by iterating*

(63) $$\begin{array}{c} q_{j}=\left(\frac{{\tilde{\beta }}_{j}}{{\tilde{\alpha }}_{j}}-1\right)\frac{1}{{\tilde{\gamma }}_{j}}\delta \left({\tilde{\beta }}_{j}-{\tilde{\alpha }}_{j}\right),\;j=1,\ldots ,k \end{array}$$

*with any initial value q≥0 converges to the globally optimal solution of the problem in Equation (62), where*

(64) $$\begin{array}{cc} {\tilde{\alpha }}_{j} & ={\left(e_{j}\right)}_{u}, \end{array}$$

(65) $$\begin{array}{cc} {\tilde{\beta }}_{j} & =a_{j}^{H}{\left(H_{j}\right)}^{-1}Z_{u}{\left(H_{j}\right)}^{-1}a_{j}, \end{array}$$

(66) $$\begin{array}{cc} {\tilde{\gamma }}_{j} & =a_{j}^{H}{\left(H_{j}\right)}^{-1}a_{j}, \end{array}$$

*with ${\left(e_{j}\right)}_{u}$ being the jth element of the vector $e_{u}$, $a_{j}$ being the jth column of the matrix A and*

(67) $$\begin{array}{c} H_{j}=\sum_{l\neq j}q_{l}a_{l}a_{l}^{H}+I_{m}. \end{array}$$

Proposition 6 introduces the nested CD inner loop of the MM1 algorithm for the problem in Equation (21), and can be proven similar to Proposition 2.

**Proposition** **7**  (The SPICE-like solver for Equation (62)). *The sequence of q generated by iterating*

(68) $$\begin{array}{cc} q & =\mathrm{sqrt}(\mathrm{diag}\left(\mathrm{Diag}\left(q\right)A^{H}Q^{-1}Z_{u}Q^{-1}A\mathrm{Diag}\left(q\right)\right)⊘e_{u}) \end{array}$$

*with any initial value q>0 converges to the globally optimal solution of the problem in Equation (62).*

The iterative procedure in Proposition 7 above is the nested SPICE-like inner loop of the MM1 algorithm for Equation (21), and can be proven similar to Proposition 3.

The MM procedure in Proposition 5 with the CD nested loop in Proposition 6 is the MM1-CD algorithm for Equation (21). The MM1-SPICE algorithm for Equation (21) is the MM procedure in Proposition 5 with the SPICE-like nested loop in Proposition 7.

#### 4.2.2. MM2 Algorithm

**Proposition** **8**  (The MM2 algorithm for Equation (21)). *The sequence $\left\{q_{u}\right\}$ generated by*

(69) $$q_{u+1}=\mathrm{sqrt}\left(\mathrm{diag}\left(\mathrm{Diag}\left(q_{u}\right)A^{H}Q_{u}^{-1}Z_{u}Q_{u}^{-1}A\mathrm{Diag}\left(q_{u}\right)\right)⊘e_{u}\right)$$

*with any initial value q>0 converges to a locally optimal solution of the problem in Equation (21).*

Due to Equation (19), by means of inserting in q the initial estimates of p and $\sigma^{2}$ given in [13], $q_{0}={\left[{\left(q_{1}\right)}_{0},\ldots ,{\left(q_{k}\right)}_{0}\right]}^{T}$ required by the iterations in Equations (62) and (69) can be

(70) $${\left(q_{j}\right)}_{0}=\frac{a_{i}^{H}\hat{R}a_{i}}{\hat{\sigma }{∥a_{i}∥}_{2}^{4}}>0,\;j=1,\ldots ,k,$$

where $\hat{R}$ is given by Equation (33) and $\hat{\sigma }$ is the mean of the first *m* smallest values of the set

(71) $$\left\{\frac{a_{1}^{H}\hat{R}a_{1}}{∥a_{1}{∥}_{2}^{2}},\ldots ,\frac{a_{k}^{H}\hat{R}a_{k}}{∥a_{k}{∥}_{2}^{2}},{\hat{R}}_{1,1},\ldots ,{\hat{R}}_{m,m}\right\}.$$

### 4.3. Discussions on the MM1 and MM2 Algorithms

The following arguments are focused on the case of spatially non-uniform noise, which are also applicable to the case of spatially uniform noise.

Both the MM1 and MM2 algorithms decrease the objective function in Equation (15) at each MM iteration and converge locally, in which the different majorizing functions are adopted. Compared to the MM2 algorithm, the MM1 algorithm has a majorizing function closer to the objective function in Equation (15) due to Equation (55). The computational burdens of the two algorithms are mainly caused by the matrix inversion operations.

Although the MM1-CD and MM1-SPICE algorithms have different nested inner iteration procedures, they converge to the same local solution theoretically because their outer MM iteration procedures are both Equation (31). Each nested inner iteration of the MM1-CD algorithm, detailed by Steps 9–17 in Algorithm 1, requires k+m−1 matrix inverse operations. In each nested inner iteration of the MM1-SPICE algorithm, presented by Steps 9–10 in Algorithm 2, only one matrix inverse operation is entailed.

It is somewhat interesting to find that the iteration of the MM2 algorithm (see Equation (56)) and the nested inner iteration of the MM1-SPICE algorithm (see Equation (45)) have similar forms. In each iteration of the MM2 algorithm, only one matrix inverse operation is needed.

### 4.4. Selection of Penalty Parameter

The penalty parameter λ in Equations (15) and (21) affects the sparsity levels of the estimates of r and q. A modified Bayesian information criterion (BIC) method [37], which is common and statistically sound, is provided here to choose an appropriate λ. Let ${\hat{r}}_{\lambda }$ and ${\hat{q}}_{\lambda }$ be the solutions of the problems in Equations (15) and (21) with a fixed λ, respectively, and denote ${\hat{W}}_{\lambda }=B\mathrm{Diag}\left({\hat{r}}_{\lambda }\right)B^{H}+J_{m}$ and ${\hat{Q}}_{\lambda }=A\mathrm{Diag}\left({\hat{q}}_{\lambda }\right)A^{H}+I_{m}$. The appropriate λ for spatially non-uniform noise and uniform noise are

(72) $$\underset{\lambda }{\arg\min}L_{0}\left({\hat{W}}_{\lambda }\right)+{\hat{z}}_{\lambda ,1}\log\left(n\right)/\left(2n\right)$$

and

(73) $$\underset{\lambda }{\arg\min}L_{0}\left({\hat{Q}}_{\lambda }\right)+{\hat{z}}_{\lambda ,2}\log\left(n\right)/\left(2n\right),$$

respectively, where ${\hat{z}}_{\lambda ,1}$ and ${\hat{z}}_{\lambda ,2}$ are the numbers of nonzero elements of the vectors ${\left({\hat{r}}_{\lambda }\right)}_{\left[1:k\right]}$ and ${\hat{q}}_{\lambda }$, respectively.

Note that the elements of ${\left({\hat{r}}_{\lambda }\right)}_{\left[1:k\right]}$ and ${\hat{q}}_{\lambda }$ can be treated as 0 if they are smaller than some certain values respectively, e.g., $\varepsilon \max({\left({\hat{r}}_{\lambda }\right)}_{\left[1:k\right]})$ and $\varepsilon \max({\hat{q}}_{\lambda })$ with a very small ε>0.

Notice that the $L_{0}\left({\hat{W}}_{\lambda }\right)$ and $L_{0}\left({\hat{Q}}_{\lambda }\right)$ in Equations (72) and (73) are substitutes for the marginal likelihood also called as Bayesian evidence required in the BIC criterion [43]. By the way, when modeling in the Bayesian framework, the marginal likelihood usually cannot be analytically calculated, but can be approximated by several computational methods in [44,45,46].

## 5. Numerical Experiments

In this section, we numerically show the performance of the proposed methods. Consider a uniform linear array (ULA), and, for each $j=1,\ldots ,k_{0}$, the steering vector corresponding to the DOA $\xi_{j}$ is

(74) $$\begin{array}{c} a\left(\xi_{j}\right)={\left[1,\exp\left(ı\pi \cos\left(\xi_{j}\right)\right),\ldots ,\exp\left(ı\pi \left(m-1\right)\cos\left(\xi_{j}\right)\right)\right]}^{T}. \end{array}$$

The array output data x(t) are generated from the model

(75) $$\begin{array}{c} x\left(t\right)=\sum_{j=1}^{k_{0}}a\left(\xi_{j}\right){\bar{s}}_{j}\left(t\right)+v\left(t\right),\;t=1,\ldots ,n, \end{array}$$

and both the source signals ${\bar{s}}_{1}\left(t\right),\ldots ,{\bar{s}}_{k_{0}}\left(t\right)$ and the observation noise v(t) are temporally independent. The SNR is defined as

(76) $$\mathrm{SNR}\left(\mathrm{dB}\right)=10\log_{10}\left(\frac{\mathrm{tr}(\bar{A}\bar{P}{\bar{A}}^{H})}{\mathrm{tr}(V)}\right),$$

where $\bar{A}=\left[a\left(\xi_{1}\right),\ldots ,a\left(\xi_{k_{0}}\right)\right]$, $\bar{P}$ is the covariance matrix of $\bar{s}\left(t\right)={\left[{\bar{s}}_{1}\left(t\right),\ldots ,{\bar{s}}_{k_{0}}\left(t\right)\right]}^{T}$, and V is the covariance matrix of v(t). The root mean-square error (RMSE) of the DOA estimate is employed to evaluate the estimation performance, which is approximated by R=500 Monte Carlo runs as

(77) $$\mathrm{RMSE}=\frac{1}{R}\sum_{i=1}^{R}\sqrt{\frac{1}{k_{0}}\sum_{j=1}^{k_{0}}{\left({\hat{\xi }}_{j,i}-\xi_{j}\right)}^{2}},$$

where ${\hat{\xi }}_{j,i}$ is the estimate of $\xi_{j}$ in the *i*th Monte Carlo run. In the following experiments, we applied the proposed methods and the SPICE [13,15] and LIKES methods [15] to estimate the DOAs. Set the grid points $\theta =\{0^{\circ },1^{\circ },\ldots ,180^{\circ }\}$, and the tolerance $\epsilon =10^{-8}$ for convergence. All methods were coded in MATLAB and executed on a workstation with two 2.10 GHz Intel Xeon CPUs.

### 5.1. Experimental Settings

#### 5.1.1. Experiment 1

Consider a scenario with Gaussian source signals and noise:

- The number of sensors in the array is m=15, and the number of snapshots is n=100.
- There are $k_{0}=4$ sources locating at $\xi_{1}=60^{\circ },\xi_{2}=80^{\circ },\xi_{3}=100^{\circ },\xi_{4}=120^{\circ }$, respectively.
- The signal $\bar{s}\left(t\right)$ is zero-mean circular complex Gaussian with covariance matrix $\bar{P}=\mathrm{Diag}\left(\left[100,100,100,100\right]\right)$.
- The noise v(t) is zero-mean circular complex Gaussian with covariance matrix $V=\sigma^{2}I_{m}$.

#### 5.1.2. Experiment 2

Consider the scenario where both the source signals and the observation noise are non-Gaussian. Let m=15. We used the experimental settings:

- The $k_{0}=4$ source signals from $\xi_{1}=10^{\circ },\;\xi_{2}=40^{\circ },\;\xi_{3}=40^{\circ }+\Delta \xi ,\;\xi_{4}=70^{\circ }$ are, respectively, as ${\bar{s}}_{1}\left(t\right)=5\exp\left(ı\varphi_{1}\left(t\right)\right),\;{\bar{s}}_{2}\left(t\right)=10\exp\left(ı\varphi_{2}\left(t\right)\right),\;{\bar{s}}_{3}\left(t\right)=10\exp\left(ı\varphi_{3}\left(t\right)\right),\;{\bar{s}}_{4}\left(t\right)=5\exp\left(ı\varphi_{4}\left(t\right)\right)$, where Δξ is the angle separation between $\xi_{2}$ and $\xi_{3}$, and $\varphi_{1}\left(t\right),\ldots ,\varphi_{4}\left(t\right)$ are independent and respectively, distributed as
  (78) $$\begin{array}{c} \begin{array}{cc} \varphi_{1}\left(t\right) & ∼2\pi \mathrm{Gamma}(1,1),\\ \varphi_{2}\left(t\right) & ∼2\pi \mathrm{Gamma}(1,1),\\ \varphi_{3}\left(t\right) & ∼U[0,2\pi ],\\ \varphi_{4}\left(t\right) & ∼U[0,2\pi ], \end{array} \end{array}$$
  where Gamma(a,b) denotes the Gamma distribution with the shape parameter *a* and scale parameter *b*, and U[a,b] denotes the uniform distribution on the interval [a,b]. Note that the source signals ${\bar{s}}_{1}\left(t\right)$, ${\bar{s}}_{2}\left(t\right)$, ${\bar{s}}_{3}\left(t\right)$ and ${\bar{s}}_{4}\left(t\right)$ have constant modulus, which is common in communication applications [13].
- The observation noise v(t) is distributed as
  (79) $$\begin{array}{c} v\left(t\right)∼\varrho n_{1}, \end{array}$$
  where ϱ∼Gamma(1,6) and $n_{1}$ is zero-mean Gaussian with covariance matrix $V=\sigma^{2}I_{m}$.

#### 5.1.3. Experiment 3

Consider a scenario with non-Gaussian source signals and non-Gaussian spatially non-uniform white noise. Let m=8 and n=100. The $k_{0}=3$ source signals locating at $\xi_{1}=40^{\circ },\xi_{2}=50^{\circ },\xi_{3}=80^{\circ }$ are ${\bar{s}}_{1}\left(t\right)=10\exp\left(ı\varphi_{2}\left(t\right)\right)$, ${\bar{s}}_{2}\left(t\right)=30\exp\left(ı\varphi_{3}\left(t\right)\right)$ and ${\bar{s}}_{3}\left(t\right)=10\exp\left(ı\varphi_{4}\left(t\right)\right)$ with $\varphi_{2}\left(t\right),\varphi_{3}\left(t\right),\varphi_{4}\left(t\right)$ being given by Equation (78). The observation noise is distributed as

(80) $$\begin{array}{c} v\left(t\right)∼\varrho n_{2}, \end{array}$$

where ϱ∼Gamma(2,2) and $n_{2}$ is zero-mean Gaussian with covariance matrix $V=\sigma^{2}\mathrm{Diag}\left(\left[m,m-1,\ldots ,1\right]\right)$.

### 5.2. Experimental Results

In Experiment 1, the MM1-CD, MM1-SPICE and MM2 algorithms with λ=0 were firstly applied. Table 1 reports the iteration numbers $n_{1},n_{2},n_{3}$, the computational time τ (in seconds), and the DOA estimation RMSEs (in degrees). Specifically, $n_{1}$ is the number of total iterations, $n_{2}$ is the number of iterations in the outer loop, amd $n_{3}$ is the average of the iterations in a nested inner loop. Besides the RMSEs, each value in Table 1 is the average of the results of 500 Monte Carlo runs. Note that, even though $n_{1}=n_{2}\times n_{3}$ in a Monte Carlo run, $n_{1}$ is not exactly equal to $n_{2}\times n_{3}$ in Table 1 because they are the average of 500 Monte Carlo runs.

As shown in Table 1, the MM1-CD and MM1-SPICE algorithms had similar RMSEs and the MM1-CD algorithm took much more time than the MM1-SPICE algorithm. Considering that the MM1-CD and MM1-SPICE algorithms theoretically converge to the identical local solution, we believe that they will always have similar RMSEs. Moreover, the MM2 algorithm had the smallest iteration numbers, the least computational time and satisfactory RMSEs. In the following, we present the DOA estimation performance only of the MM1-SPICE and MM2 algorithms. Note that the MM1-SPICE and MM2 algorithms may converge to different local solutions, and then they are referred to as two different estimation methods hereinafter. Specifically, we compared the following six methods:

1. LIKES: The ML method proposed in [15] under the assumption that the array output x(t) is Gaussian distributed.
2. SPICE: A sparse covariance-based estimation method proposed in [13] with no distribution assumption.
3. MM1-SPICE-0: The MM1-SPICE method with the penalty parameter λ=0;
4. MM1-SPICE-P: The MM1-SPICE method with the penalty parameter selected by the criterion in Section 4.4.
5. MM2-0: The MM2 method with the penalty parameter λ=0.
6. MM2-P: The MM2 method with the penalty parameter selected by the criterion in Section 4.4.

It is worth presenting that in, Experiments 1–3, the standard MUSIC algorithm was found to be almost ineffective, thus we do not illustrate it.

**Figure 1:**
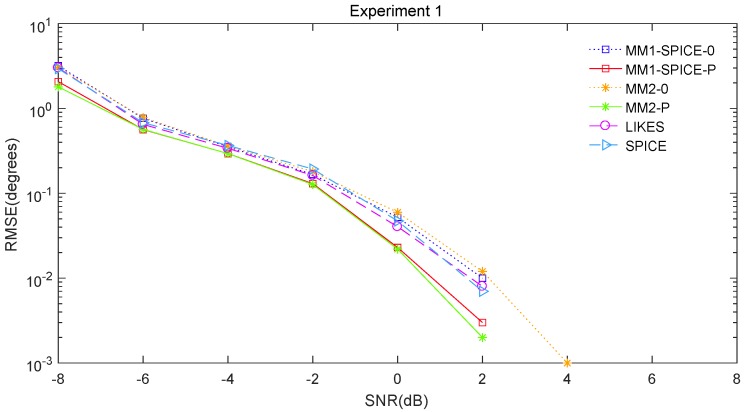
DOA estimation RMSE versus SNR for Experiment 1.

**Figure 2:**
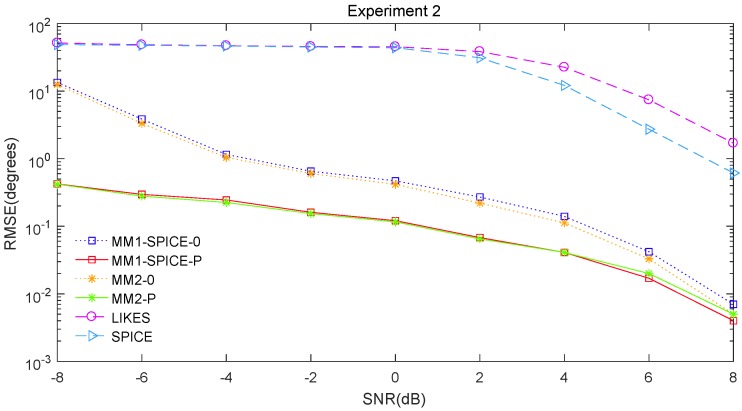
DOA estimation RMSE versus SNR for Experiment 2 with n=100 and $\Delta \xi =15^{\circ }$.

**Figure 3:**
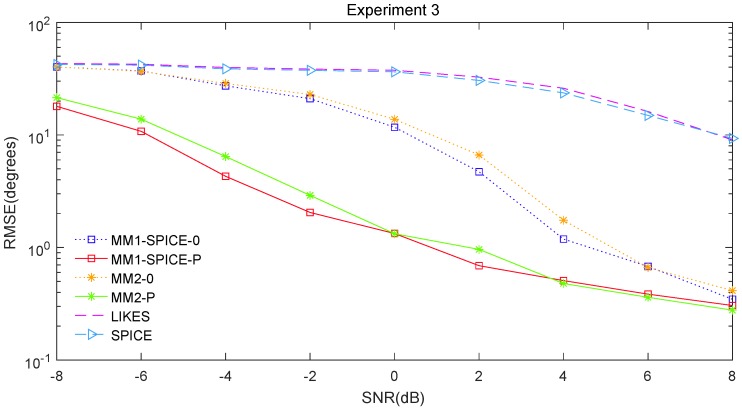
DOA estimation RMSE versus SNR for Experiment 3.

**Figure 4:**
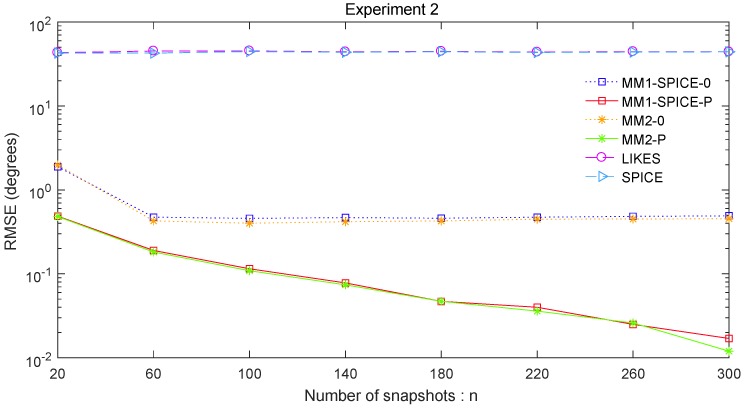
DOA estimation RMSE versus the number of snapshots *n* for Experiment 2 with SNR=0dB and $\Delta \xi =15^{\circ }$.

**Figure 5:**
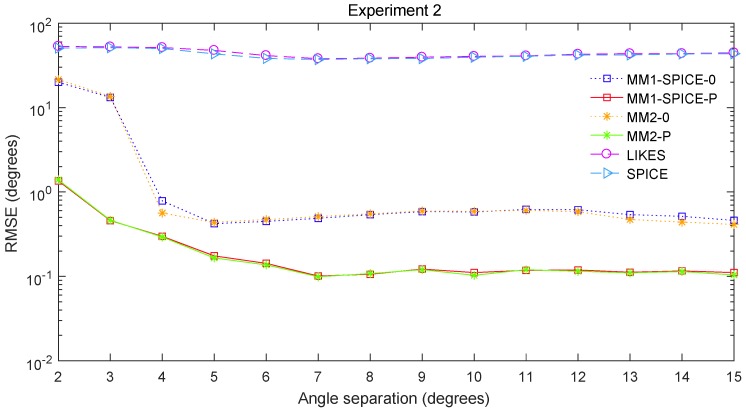
DOA estimation RMSE versus the angle separation Δξ for Experiment 2 with n=100 and SNR=0dB.

The RMSE comparisons of the above six methods are illustrated in Figure 1, Figure 2 and Figure 3 for Experiments 1–3 with different SNRs, respectively. Figure 4 and Figure 5 for Experiment 2 show the DOA estimation RMSEs of the above six methods versus the number of snapshots and the angle separation, respectively.

In Figure 1, we can see that the MM1-SPICE-0 and MM2-0 methods were comparable to the LIKES and SPICE methods in the Gaussian cases. As shown in Figure 2, Figure 3, Figure 4 and Figure 5, the scenarios of non-Gaussian random source signals and heavy-tailed random noise, the MM1-SPICE-0 and MM2-0 methods performed much better than the LIKES and SPICE methods. In other words, the ML methods designed for the CES outputs were effective in the simulation scenarios of Gaussian and non-Gaussian distributions.

As shown in Figure 1, Figure 2, Figure 3, Figure 4 and Figure 5, we found that the penalized ML methods, i.e., the MM1-SPICE-P and MM2-P methods, had smaller RMSEs than the other four methods. As shown in Figure 4, with the increase of the number of snapshots, the performance of the MM1-SPICE-P and MM2-P methods improved but the performance of the MM1-SPICE-0 and MM2-0 methods remained virtually unchanged. Figure 5 shows that, as Δξ increased from $2^{\circ }$ to $6^{\circ }$, the performance of the MM1-SPICE-P and MM2-P methods became better, while, when Δξ was larger than $4^{\circ }$, the performance of the MM1-SPICE-0 and MM2-0 methods no longer improved. As shown in Figure 1, Figure 2 and Figure 3, unsurprisingly, as the SNR increased, the performance of all the six methods became better.

For illustrating the difference between the penalized and un-penalized ML methods, we evaluated the normalized spectrum (NS):

(81) $$\begin{array}{c} \mathrm{NS}=\frac{{\hat{r}}_{\left[1:k\right]}}{\max({\hat{r}}_{\left[1:k\right]})}\;\mathrm{or}\;\frac{\hat{q}}{\max(\hat{q})}, \end{array}$$

where $\hat{r}$ and $\hat{q}$ are, respectively, the estimates of r and q.

**Figure 6:**
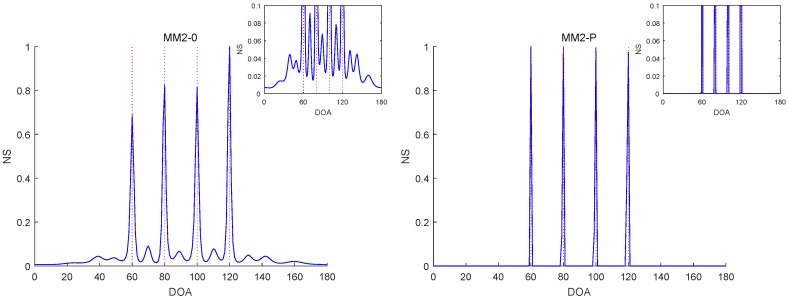
NSs of Experiment 1 with SNR=0dB.

**Figure 7:**
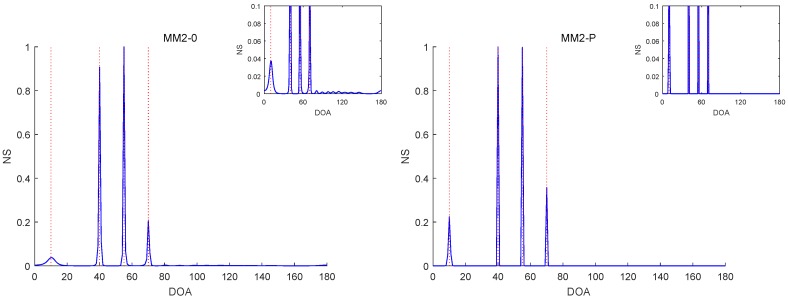
NSs of Experiment 2 with n=100, SNR=0dB and $\Delta \xi =15^{\circ }$.

**Figure 8:**
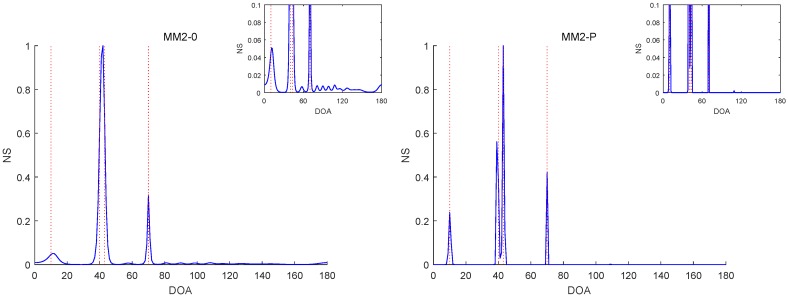
NSs of Experiment 2 with n=100, SNR=0dB and $\Delta \xi =3^{\circ }$.

**Figure 9:**
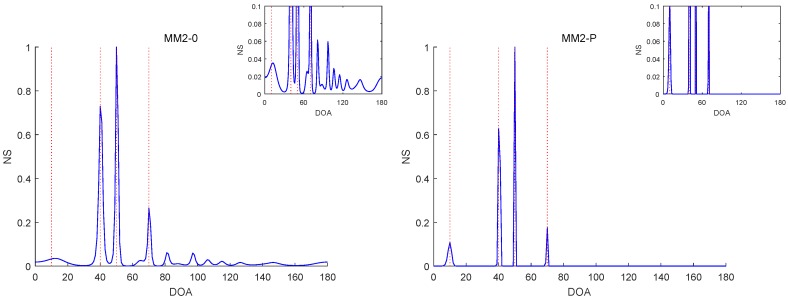
NSs of Experiment 2 with n=20, SNR=−10dB and $\Delta \xi =10^{\circ }$.

**Figure 10:**
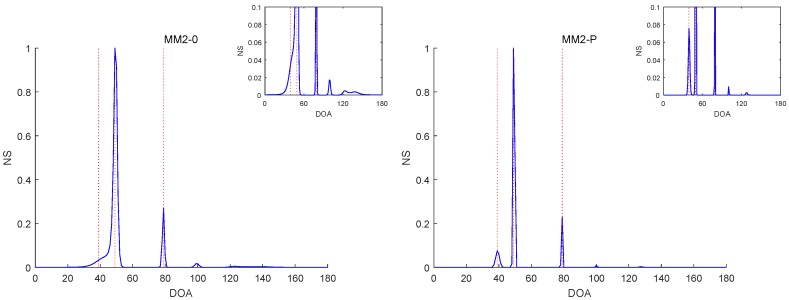
NSs of Experiment 3 with SNR=0dB.

Figure 6, Figure 7, Figure 8, Figure 9 and Figure 10 report the NSs of Experiments 1–3 by the MM2-0 and MM2-P methods (the curves of the NSs of the MM-SPICE-0 and MM2-SPICE-P methods are similar to those of the MM2-0 and MM2-P methods, respectively). Figure 6 and Figure 10 are for Experiments 1 and 3 with SNR=0dB, respectively. Figure 7 and Figure 8 are both for Experiment 2 with n=100 and SNR=0dB, but they involve two scenarios with different angle separations. Particularly, Figure 8 is for the very small $\Delta \xi =3^{\circ }$, as opposed to Figure 7 for $\Delta \xi =15^{\circ }$. Figure 9 is also for Experiment 2, but it is about the scenario with small snapshot size n=20, small SNR=−10dB and moderate angle separation $\Delta \xi =10^{\circ }$. Note that the red dashed lines mark the true DOAs and the NS curves in each figure are the results of a randomly selected realization. We can clearly see in Figure 6, Figure 7, Figure 8, Figure 9 and Figure 10 that the MM2-P method having the $l_{1}$-norm penalty added yielded a higher angular resolution.

## 6. Conclusions

This paper provides a penalized ML method for DOA estimation under the assumption that the array output has a CES distribution. Two MM algorithms, named MM1 and MM2, are developed for solving the non-convex penalized ML optimization problem for spatially uniform or non-uniform noise. They converge locally with different rates. The numerical experiments showed that the MM2 ran faster and performed as well as the MM1 when estimating the DOAs. Their rates of convergence will be further explored theoretically in future.

It is worth mentioning that, in numerical simulations, the proposed $l_{1}$-norm penalized likelihood method effectively estimated the DOAs, although the values of nonzero elements of r or p were not estimated very accurately. By replacing the $l_{1}$-norm penalty by other proper penalties (e.g., the smoothly clipped absolute deviation (SCAD) [47], adaptive LASSO [48], or $l_{p}$-norm penalty (0≤p<1)), more accurate estimates of the unknown parameter may be derived in the future.

If the $l_{1}$-norm penalty is replaced by the adaptive LASSO penalty, then the algorithms proposed in this paper can be applied almost without modification because the adaptive LASSO penalty is a weighted $l_{1}$-norm penalty. When the non-convex SCAD or the $l_{p}$-norm penalty (0≤p<1) is employed, the convex majorizing functions given in [40,49] can be exploited based on the MM framework, and then the algorithms in this paper with minor modifications are also applicable.

When we have some informative prior knowledge on the directions of source signals, the problem of estimating the DOAs can be formulated as maximizing a sparse posterior likelihood from the Bayesian perspective. The sparse Bayesian method of estimating DOAs from the CES array outputs is interesting and is worth studying, while how to formulate and solve a sparse posterior likelihood optimization and how to do model selection are challenges. The BIC criterion can be used for the model selection, in which the Bayesian evidence difficult to be analytically derived can be approximated by the methods in [44,45,46]. More research along this line will be done in the future.

## Figures and Tables

**Table 1:** Comparison of computational complexities and RMSEs.

SNR	Method	$n_{1}$	$n_{2}$	$n_{3}$	τ	RMSE
−1dB	MM1-CD	525.5600	16.0440	32.7550	27.9710	0.0877
	MM1-SPICE	317.6500	16.9280	18.7370	0.3805	0.0926
	MM2	129.5000	−	−	0.3266	0.1059
−3dB	MM1-CD	539.2700	16.0000	33.7040	28.7700	0.2345
	MM1-SPICE	310.0300	16.6260	18.6070	0.3714	0.2434
	MM2	129.3800	−	−	0.3260	0.2568
−5dB	MM1-CD	561.0900	16.4380	34.1500	29.3910	0.4292
	MM1-SPICE	303.1400	16.3340	18.5130	0.3590	0.4681
	MM2	129.1400	−	−	0.3195	0.4840

## References

[1] Schmidt R.O. (1986). Multiple emitter location and signal parameter estimation. IEEE Trans. Antennas Propag..

[2] Roy R., Kailath T. (1989). ESPRIT-estimation of signal parameters via rotational invariance techniques. IEEE Trans. Acoust. Speech Signal Process..

[3] Stoica P., Sharman K.C. (1990). Maximum likelihood methods for direction-of-arrival estimation. IEEE Trans. Acoust. Speech Signal Process..

[4] Friedlander B. (1993). The root-MUSIC algorithm for direction finding with interpolated arrays. Signal Process..

[5] Yang Z., Li J., Stoica P., Xie L., Chellappa R., Theodoridis S. (2018). Sparse methods for direction-of-arrival estimation. Academic Press Library in Signal Processing, Volume 7: Array, Radar and Communications Engineering.

[6] Gorodnitsky I.F., Rao B.D. (1997). Sparse signal reconstruction from limited data using FOCUSS: A re-weighted minimum norm algorithm. IEEE Trans. Signal Process..

[7] Tipping M.E. (2001). Sparse Bayesian learning and the relevance vector machine. J. Mach. Learn. Res..

[8] Wipf D.P., Rao B.D. (2004). Sparse Bayesian learning for basis selection. IEEE Trans. Signal Process..

[9] Malioutov D., Çetin M., Willsky A.S. (2005). A sparse signal reconstruction perspective for source localization with sensor arrays. IEEE Trans. Signal Process..

[10] Babacan S.D., Molina R., Katsaggelos A.K. (2010). Bayesian compressive sensing using Laplace priors. IEEE Trans. Image Process..

[11] Hyder M.M., Mahata K. (2010). Direction-of-arrival estimation using a mixed *l*_2,0_ norm approximation. IEEE Trans. Signal Process..

[12] Xu X., Wei X., Ye Z. (2012). DOA estimation based on sparse signal recovery utilizing weighted *l*_1_-norm penalty. IEEE Signal Process. Lett..

[13] Stoica P., Babu P., Li J. (2011). SPICE: A sparse covariance-based estimation method for array processing. IEEE Trans. Signal Process..

[14] Stoica P., Babu P., Li J. (2011). New method of sparse parameter estimation in separable models and its use for spectral analysis of irregularly sampled data. IEEE Trans. Signal Process..

[15] Stoica P., Babu P. (2012). SPICE and LIKES: Two hyperparameter-free methods for sparse-parameter estimation. Signal Process..

[16] Stoica P., Zachariah D., Li J. (2014). Weighted SPICE: A unifying approach for hyperparameter-free sparse estimation. Digit. Signal Process..

[17] Yin J., Chen T. (2011). Direction-of-arrival estimation using a sparse representation of array covariance vectors. IEEE Trans. Signal Process..

[18] Zheng J., Kaveh M. (2013). Sparse spatial spectral estimation: A covariance fitting algorithm, performance and regularization. IEEE Trans. Signal Process..

[19] Liu Z.M., Huang Z.T., Zhou Y.Y. (2013). Array signal processing via sparsity-inducing representation of the array covariance matrix. IEEE Trans. Aerosp. Electron. Syst..

[20] He Z.Q., Shi Z.P., Huang L. (2014). Covariance sparsity-aware DOA estimation for nonuniform noise. Digit. Signal Process..

[21] Chen T., Wu H., Zhao Z. (2016). The real-valued sparse direction of arrival (DOA) estimation based on the Khatri-Rao product. Sensors.

[22] Wang Y., Hashemi-Sakhtsari A., Trinkle M., Ng B.W.H. (2018). Sparsity-aware DOA estimation of quasi-stationary signals using nested arrays. Signal Process..

[23] Shutin D., Fleur B.H. (2011). Sparse variational Bayesian SAGE algorithm with application to the estimation of multipath wireless channels. IEEE Trans. Signal Process..

[24] Hu L., Shi Z., Zhou J., Fu Q. (2012). Compressed sensing of complex sinusoids: An approach based on dictionary refinement. IEEE Trans. Signal Process..

[25] Yang Z., Zhang C., Xie L. (2012). Robustly stable signal recovery in compressed sensing with structured matrix perturbation. IEEE Trans. Signal Process..

[26] Yang Z., Xie L., Zhang C. (2013). Off-grid direction of arrival estimation using sparse Bayesian inference. IEEE Trans. Signal Process..

[27] Fan Y., Wang J., Du R., Lv G. (2018). Sparse method for direction of arrival estimation using denoised fourth-order cumulants vector. Sensors.

[28] Wu X., Zhu W.P., Yan J., Zhang Z. (2018). Two sparse-based methods for off-grid direction-of-arrival estimation. Signal Process..

[29] Bhaskar B.N., Tang G., Recht B. (2013). Atomic norm denoising with applications to line spectral estimation. IEEE Trans. Signal Process..

[30] Yang Z., Xie L., Zhang C. (2014). A discretization-free sparse and parametric approach for linear array signal processing. IEEE Trans. Signal Process..

[31] Yang Z., Xie L. (2016). Enhancing sparsity and resolution via reweighted atomic norm minimization. IEEE Trans. Signal Process..

[32] Wang X., Wang W., Li X., Liu Q., Liu J. (2016). Sparsity-aware DOA estimation scheme for noncircular source in MIMO Radar. Sensors.

[33] Jing X., Liu X., Liu H. (2017). A sparse recovery method for DOA estimation based on the sample covariance vectors. Circuits Syst. Signal Process..

[34] Stoica P., Babu P. (2012). Sparse estimation of spectral lines: Grid selection problems and their solutions. IEEE Trans. Signal Process..

[35] Ollila E., Tyler D.E., Koivunen V., Vincent H. (2012). Complex elliptically symmetric distributions: Survey, new results and applications. IEEE Trans. Signal Process..

[36] Sun Y., Babu P., Palomar D.P. (2016). Robust estimation of structured covariance matrix for heavy-tailed elliptical distributions. IEEE Trans. Signal Process..

[37] Pourahmadi M. (2013). High-Dimensional Covariance Estimation.

[38] Sangston K.J., Gini F., Greco M.S. (2012). Coherent radar target detection in heavy-tailed compound-Gaussian clutter. IEEE Trans. Aerosp. Electron. Syst..

[39] Hunter D.R., Lange K. (2004). A tutorial on MM algorithms. Am. Stat..

[40] Sun Y., Babu P., Palomar D.P. (2017). Majorization-Minimization algorithms in signal processing, communications, and machine learning. IEEE Trans. Signal Process..

[41] Kent J.T., Tyler D.E. (1988). Maximum likelihood estimation for the wrapped Cauchy distribution. J. Appl. Stat..

[42] Luo Z.Q., Tseng P. (1992). On the convergence of the coordinate descent method for convex differentiable minimization. J. Optim. Theory Appl..

[43] Schwarz G. (1978). Estimating the dimension of a model. Ann. Stat..

[44] Friel N., Wyse J. (2012). Estimating the evidence—A review. Stat. Neerl..

[45] Martino L., Elvira V., Luengo D., Corander J. (2015). An adaptive population importance sampler: Learning from uncertainty. IEEE Trans. Signal Process..

[46] Martino L., Elvira V., Luengo D., Corander J. (2017). Layered adaptive importance sampling. Stat. Comput..

[47] Fan J., Li R. (2001). Variable selection via nonconcave penalized likilihood and its oracle properties. J. Am. Stat. Assoc..

[48] Zou H. (2006). The adaptive Lasso and its oracle properties. J. Am. Stat. Assoc..

[49] Fan J., Feng Y., Wu Y. (2009). Network exploration via the adaptive LASSO and SCAD penalties. Ann. Appl. Stat..

